# Outcomes After Major Surgical Procedures in Octogenarians: A Nationwide Cohort Study

**DOI:** 10.1007/s00268-022-06642-6

**Published:** 2022-08-04

**Authors:** Arthur K. E. Elfrink, Anna J. Alberga, Mark I. van Berge Henegouwen, Wilhelmina H. Scheurs, Lydia G. M. van der Geest, Hence J. M. Verhagen, Jan-Willem T. Dekker, Dirk J. Grünhagen, Michel W. J. M. Wouters, Joost M. Klaase, DICA Study Group, Daan M. Voeten, J. Annelie Suurmeijer, Anne-Loes Warps, Lisa van der Woude, Robin Detering, Nienke Wolfhagen

**Affiliations:** 1 Scientific Bureau, Dutch Institute for Clinical Auditing, 2333 AA Leiden, The Netherlands; 2 Department of Hepatobiliary Surgery and Liver Transplantation, Universitair Medisch Centrum Groningen, Groningen, The Netherlands; 3 Department of Vascular Surgery, Erasmus MC, Rotterdam, The Netherlands; 4 Department of Surgery, Amsterdam UMC, Locatie AMC, Cancer Center Amsterdam, Universiteit Van Amsterdam, Amsterdam, The Netherlands; 5 Department of Surgery, Noordwest Ziekenhuisgroep, Alkmaar, The Netherlands; 6 Integraal Kankercentrum Nederland, Utrecht, The Netherlands; 7 Department of Surgery, Reinier de Graaf Gasthuis, Delft, The Netherlands; 8 Department of Surgery, Antoni Van Leeuwenhoek, Amsterdam, The Netherlands

## Abstract

**Introduction:**

Aging of the worldwide population has been observed, and postoperative outcomes could be worse in elderly patients. This nationwide study assessed trends in number of surgical resections in octogenarians regarding various major surgical procedures and associated postoperative outcomes.

**Methods:**

All patients who underwent surgery between 2014 and 2018 were included from Dutch nationwide quality registries regarding esophageal, stomach, pancreas, colorectal liver metastases, colorectal cancer, lung cancer and abdominal aortic aneurysms (AAA). For each quality registry, the number of patients who were 80 years or older (octogenarians) was calculated per year. Postoperative outcomes were length of stay (LOS), 30 day major morbidity and 30 day mortality between octogenarians and younger patients.

**Results:**

No increase in absolute number and proportion of octogenarians that underwent surgery was observed. Median LOS was higher in octogenarians who underwent surgery for colorectal cancer, colorectal liver metastases, lung cancer, pancreatic disease and esophageal cancer. 30 day major morbidity was higher in octogenarians who underwent surgery for colon cancer, esophageal cancer and elective AAA-repair. 30 day mortality was higher in octogenarians who underwent surgery for colorectal cancer, lung cancer, stomach cancer, pancreatic disease, esophageal cancer and elective AAA-repair. Median LOS decreased between 2014 and 2018 in octogenarians who underwent surgery for stomach cancer and colorectal cancer. 30 day major morbidity decreased between 2014 and 2018 in octogenarians who underwent surgery for colon cancer. No trends were observed in octogenarians regarding 30 day mortality between 2014 and 2018.

**Conclusion:**

No increase over time in absolute number and proportion of octogenarians that underwent major surgery was observed in the Netherlands. Postoperative outcomes were worse in octogenarians.

**Supplementary Information:**

The online version contains supplementary material available at 10.1007/s00268-022-06642-6.

## Introduction

Aging of the population in Western countries has been described in the last 20 years which might be accompanied by a higher incidence of surgical procedures in the elderly [1]. In the Netherlands, the proportion of population who were 80 years or older (octogenarians) increased from 3.2% in 2000 to 4.6% in 2019 [2]. Major surgical procedures were described to be performed more often in octogenarians after the year 2000 [3].

As people get older, an increase of the number comorbidities of a patient scheduled for surgery has been described [3]. Having multiple comorbidities is common in elderly patients and is estimated to be 80% in octogenarians [4, 5]. Comorbidities are a risk factor for occurrence of postoperative morbidity and mortality due to a decrease of functional reserve [6–8]. Several studies from various surgical fields on major surgical procedures in elderly patients have shown higher morbidity and mortality rates [3, 9–12]. Clinical studies have also shown that higher age and additional comorbidities of a patient can be a reason to refrain from major surgical procedures as occurrence of perioperative complications can decrease quality of life and long-term outcomes [13].

To improve quality of care for octogenarians, insights into current daily practice and outcomes are needed. Improvement measures to improve quality of care and patient selection before proceeding to major surgical procedures can be based on current outcomes. To date, it is unclear whether aging of the population leads to an increase in octogenarians who undergo major surgical procedures in the Netherlands. Also, nationwide assessment of postoperative outcomes in octogenarians who undergo major surgical procedures in the Netherlands is lacking.

The aim of this nationwide study is to assess trends in the number and proportion of octogenarians undergoing major surgical procedures and their associated postoperative outcomes over time.

## Methods

### Study Population

Patients were included using the prospective quality registries for several indications for surgery from the Dutch Institute for Clinical Auditing (DICA): the Dutch Institute for Clinical Auditing (DICA): the Dutch Upper GI Cancer Audit (DUCA), the Dutch Pancreatic Cancer Audit (DPCA), the Dutch HepatoBiliary Audit (DHBA), the Dutch ColoRectal Audit (DCRA), the Dutch Lung Cancer Audit-Surgery (DLCA-S) and the Dutch Surgical Aneurysm Audit (DSAA) [14]. The number of patients diagnosed with oncological conditions was retrieved using data from the Dutch cancer registry (NKR). These data were available until 2018 for esophageal, stomach, colorectal and lung cancer [14, 15].

All patients who were 18 year or older and underwent primary surgery from the several quality registries between the 1st of January 2014 and 31st of December 2018 were included. From the DUCA, DPCA, DHBA, DCRA and DLCA-S, patients were included who underwent resection of esophageal or stomach cancer, resection for benign or malignant pancreatic disease, colorectal liver metastases (CRLM), colorectal cancer, or lung cancer, respectively. From the DSAA, patients who underwent elective repair of an abdominal aneurysm (AAA) were included.

Patients were divided for analyses in two groups: patients who were younger than 80 years (80-) or those who were 80 years or older (octogenarians).

For this study, no ethical approval was needed under Dutch Law. However, clinical audit boards of each quality registry approved this study. This study was performed according to the Strobe guidelines for cohort studies [16] (Appendix A).

### Outcomes

Primary outcomes were postoperative outcomes which consisted of length of hospital stay (LOS), 30 day major morbidity defined as complications graded Clavien–Dindo 3a or higher, and 30 day mortality [17]. If complications were not scored according to the Clavien–Dindo classification in a registry, a postoperative complicated course was used. This composite outcome is defined as a complication accompanied by either one of the three: prolonged LOS (> 14 days), reintervention or death within 30 days of surgery [18–20]. This outcome was used for the DLCA, DCRA and DSAA. All outcomes were compared between patients who were younger than 80 years and octogenarians.

Secondary outcomes were trends in number of patients over the years who were younger than 80 per quality registry compared to octogenarians over the years, and postoperative outcomes.

### Statistical Analyses

Categorical variables were displayed as appropriate using numbers accompanied by percentages. Continuous variables were displayed as mean accompanied with standard deviation (SD) in case of normal distribution. If non-normal distribution of data was observed, the median was shown accompanied by interquartile ranged (IQR).

Postoperative outcomes were compared between the groups using the Chi-squared test in case of dichotomous outcomes or Mann–Whitney U test in case of continuous outcomes. Analysis regarding the number of octogenarians within quality registries compared to total number of patients over the years and compared to the total number of diagnosed patients with the same oncological condition was performed using linear regression analysis.

For trends in postoperative outcomes, trend analysis over time were performed using linear or logistic regression as appropriate for continuous or dichotomous outcomes. The beta coefficient (ß) or odds ratio (OR), including 95% confidence intervals, was displayed as influence per year if a trend was observed.

In all analyses, a two-sided *p*-value < 0.05 was seen as significant.

## Results

The total included number of patients per quality registry ranged from 2509 to 38,229 patients (Table 1). The percentage of octogenarians ranged between quality registries between 3.5% (esophageal cancer) and 21.4% (AAA). The percentage of octogenarians in oncological quality registries compared to the total number of octogenarians diagnosed with cancer ranged between 4.6% (lung cancer) and 73.7% (colon cancer).

**Table 1:** Number of patients stratified for age categories per year for every quality registry

Type of surgery	2014	2015	2016	2017	2018	Total	*p*-value trend
*Abdominal aortic aneurysm*							
*N*—total	2542	2464	2564	2488	2528	12,586	
*N*—80 + total	553	509	540	536	552	2690	0.720
% 80 +	21.8	20.7	21.1	21.5	21.8	21.4	0.586
Mean age (SD)	73.3 (7.6)	73.2 (7.6)	73.2 (7.5)	73.2 (7.5)	73.4 (7.7)	73.2 (7.7)	0.626
*Colon cancer*							
*N*—total	7783	8470	8244	7094	6608	38,199	
*N*—80 + total	1562	1496	1515	1421	1324	7318	**0.019**
% 80 +	20.1	17.7	18.4	20.0	20.0	19.2	0.600
Mean age (SD)	70.9 (10.4)	69.8 (10.1)	70.0 (10.2)	70.3 (10.6)	70.3 (10.9)	70.2 (10.4)	0.662
*Colorectal liver metastases*							
*N*—total	771	884	1008	1009	838	4510	
*N*—80 + total	56	62	64	82	50	314	0.867
% 80 +	7.3	7.0	6.3	8.1	6.0	7.0	0.649
Mean age (SD)	65.5 (10.1)	66.1 (10.0)	65.8 (10.4)	66.3 (10.5)	65.2 (10.6)	65.7 (10.7)	0.838
*Lung cancer*							
*N*—total	1507	1631	1907	2070	2241	9356	
*N*—80 + total	92	93	91	100	126	502	0.101
% 80 +	6.1	5.7	4.8	4.8	5.6	5.4	0.384
Mean age (SD)	66.0 (9.4)	66.1 (9.0)	65.9 (9.5)	66.4 (9.0)	66.8 (9.1)	66.3 (9.9)	0.077
*Stomach cancer*							
*N*—total	578	485	552	441	453	2509	
*N*—80 + total	86	82	109	89	80	446	0.913
% 80 +	14.9	16.9	19.7	20.2	17.7	17.8	0.240
Mean age (SD)	69.0 (10.9)	70.0 (42.4)	69.3 (11.5)	69.6 (11.5)	69.7 (11.7)	69.9 (11.5)	0.140
Pancreas resection							
*N*—total	871	870	880	945	904	4470	
*N*—80 + total	53	48	58	69	66	294	0.069
% 80 +	6.1	5.5	6.6	7.3	7.3	6.6	0.063
Mean age (SD)	64.8 (11.8)	65.0 (11.7)	65.6 (11.0)	65.2 (11.8)	65.9 (11.6)	65.3 (11.6)	0.074
Rectal cancer							
*N*—total	2957	3280	3046	3063	2644	15,005	
*N*—80 + total	365	368	345	401	329	1808	0.713
% 80 +	12.3	11.2	11.3	13.1	12.4	12.0	0.491
Mean age (SD)	67.8 (10.6)	67.4 (10.0)	67.0 (10.2)	67.3 (10.6)	67.1 (10.8)	67.3 (10.4)	0.201
Esophageal cancer							
*N*—total	770	837	793	820	799	4019	
*N*—80 + total	28	38	24	26	26	142	0.440
% 80 +	3.6	4.5	3.0	3.2	3.3	3.5	0.334
Mean age (SD)	65.2 (9.0)	65.3 (8.8)	65.3 (8.4)	65.8 (8.5)	66.3 (8.7)	65.8 (8.9)	**0.022**

Bold values are statistically significant (*p* < 0.05)

*N* number of patients, *SD* standard deviation

### Trends in Number of Octogenarians

The mean age ranged between 65.3 and 73.2 years for the different indications for surgery. An increasing number of older patients that underwent resection of esophageal cancer were observed with a mean age of 65.2 years in 2014 and 66.3 years in 2018 (*p* = 0.022). In other quality registries, no significant increase in mean age was observed.

**Fig. 1:**
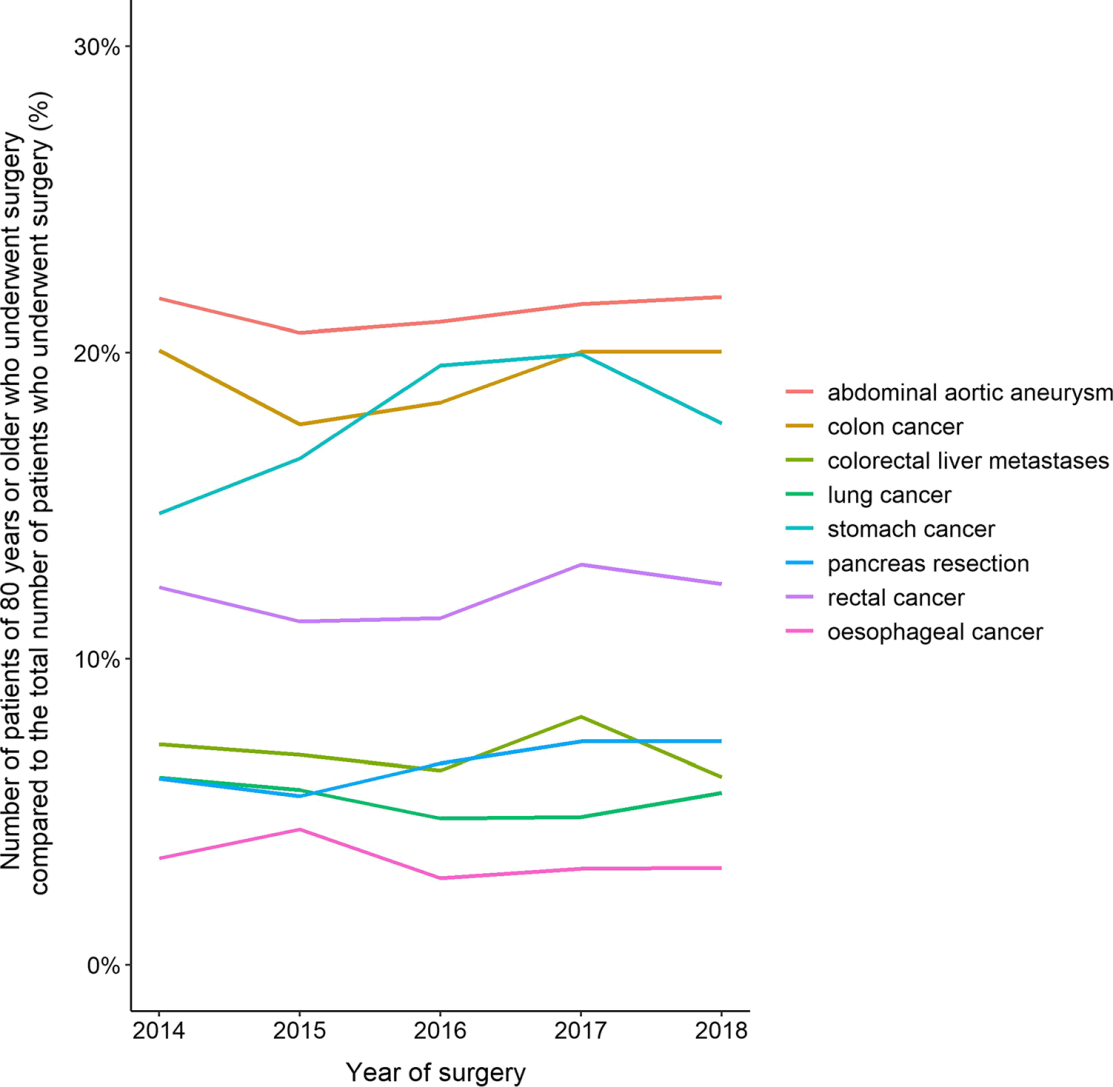
Trend in number of patients of 80 years or older who underwent surgery per quality registry compared to the total number of patients who underwent surgery per quality registry per year

No increase in the proportion of octogenarians was observed for esophageal cancer, CRLM, colon cancer, rectal cancer, lung cancer and abdominal aortic aneurysms (Table 1, Fig. 1). The proportion of octogenarians who underwent pancreas resection slightly increased from 6.1% in 2014 to 7.3% in 2018 (*p* = 0.063).

The proportion of octogenarians in oncological quality registries did not increase compared to the Dutch cancer registry (supplementary Fig. 1, supplementary table 1).

### Postoperative Outcomes

**Fig. 2:**
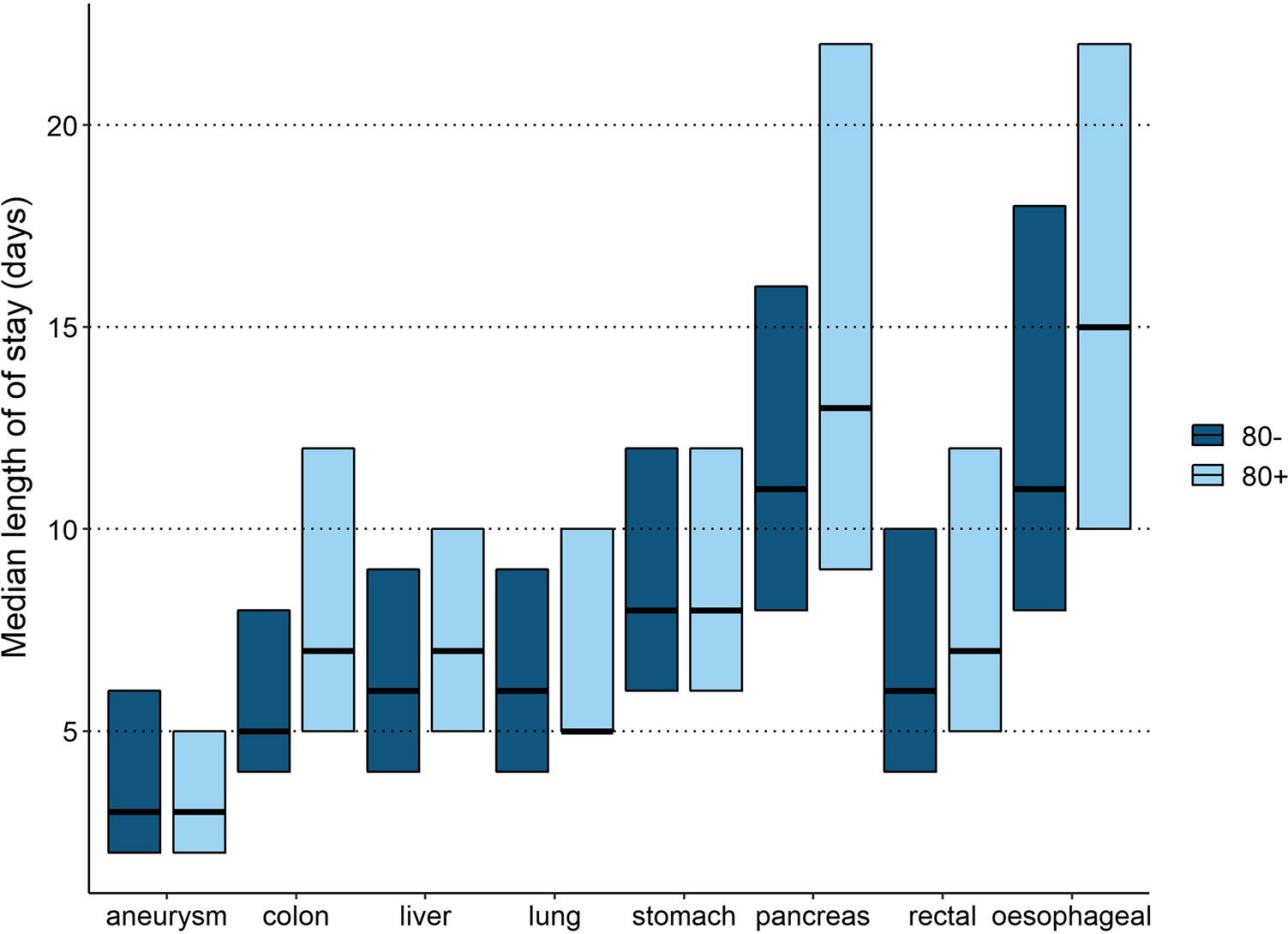
Median length of stay with interquartile range per type of surgery stratified for patients who were younger than 80 years versus octogenarians

Median LOS was longer in octogenarians who underwent surgery for colon cancer compared to non-octogenarians (7 days (IQR 5–12) vs. 5 days (IQR 4–8), *p* < 0.001), CRLM (7 days (IQR 5–10) vs. 6 days (IQR 4–9), *p* < 0.001), lung cancer (7 days (IQR 5–10) vs. 6 days (IQR 4–9)), *p* < 0.001), pancreatic disease 13 days (IQR 9–22) vs. 11 days (IQR 8–16), *p* < 0.001), rectal cancer (7 days (IQR 5–12) vs. 6 days (IQR 4–10), *p* < 0.001), esophageal cancer (15 days (IQR 10–22) vs. 11 days (IQR 8–18), *p* < 0.001) as compared to younger patients. Median LOS in octogenarians who underwent AAA-repair was shorter compared to non-octogenarians (3 (IQR 2–5) vs. 3 (IQR 2–6), *p* = 0.046). This difference was due to more endovascular AAA-repair in octogenarians. For stomach cancer, no difference in median LOS was observed (Table 2 and Fig. 2).

**Table 2:** Postoperative outcomes stratified for age per quality registry

Type of surgery	< 80 years	≥ 80 years	RR	*p*-value
	Median (IQR)/*N* (%)	Median (IQR)/*N* (%)		
Abdominal aortic aneurysm	9896 (78.6)	2690 (21.4)		
Length of stay	3 (2–6)	3 (2–5)		**0.046**
30 day major morbidity	1207 (12.2)	368 (13.7)	1.12	0.045
30 day mortality	159 (1.6)	59 (2.2)	1.37	**0.043**
Colon cancer	30881 (80.8)	7318 (19.2)		
Length of stay	5 (4–8)	7 (5–12)		** < 0.001**
30 day major morbidity	4211 (13.6)	1568 (21.4)	1.57	** < 0.001**
30 day mortality	449 (1.5)	425 (5.8)	3.99	** < 0.001**
Colorectal liver metastases	4196 (93.0)	314 (7.0)		
Length of stay	6 (4–9)	7 (5–10)		** < 0.001**
30 day major morbidity	360 (8.6)	37 (11.8)	1.37	0.067
30 day mortality	59 (1.4)	6 (1.9)	1.36	0.632
Lung cancer	8836 (94.6)	502 (5.4)		
Length of stay	6 (4–9)	7 (5–10)		** < 0.001**
30 day major morbidity	791 (9.0)	52 (10.4)	1.16	0.322
30 day mortality	162 (1.8)	28 (5.6)	3.05	** < 0.001**
Stomach cancer	2063 (82.2)	446 (17.8)		
Length of stay	8 (6–12)	8 (6–12)		0.527
30 day major morbidity	360 (17.5)	76 (17.0)	0.90	0.890
30 day mortality	96 (4.7)	34 (7.6)	1.64	**0.037**
Pancreas resection	4176 (93.4)	294 (6.6)		
Length of stay	11 (8–16)	13 (9–22)		** < 0.001**
30 day major morbidity	1151 (27.6)	86 (29.3)	1.06	0.434
30 day mortality	127 (3.0)	17 (5.8)	1.90	**0.016**
Rectal cancer	13182 (88.0)	1808 (12.0)		
Length of stay	6 (4–10)	7 (5–12)		** < 0.001**
30 day major morbidity	2594 (19.7)	370 (20.5)	1.04	0.450
30 day mortality	101 (0.8)	70 (3.9)	5.06	** < 0.001**
Esophageal cancer	3877 (96.5)	142 (3.5)		
Length of stay	11 (8–18)	15 (10–22)		** < 0.001**
30 day major morbidity	1118 (28.8)	56 (39.4)	1.37	**0.008**
30 day mortality	115 (3.0)	12 (8.5)	2.85	**0.001**

Bold values are statistically significant (*p* < 0.05)

*RR* relative risk

*p*-value is for statistical significance of percentage difference between < 80 and 80 years or older

30 day major morbidity was higher in octogenarians who underwent surgery for AAA (13.7 vs. 12.2%, *p* = 0.045), colon cancer (21.4% vs. 13.6%, *p* < 0.001) and esophageal cancer (39.4% vs 28.8%, *p* = 0.008) (Table 2).

Thirty-day mortality was higher in octogenarians who underwent surgery for AAA (2.2% vs. 1.6%, *p* = 0.043), colon cancer (5.8% vs 1.5%, *p* < 0.001), lung cancer (5.6% vs 1.8%, *p* < 0.001), stomach cancer (7.6% vs 4.7%, *p* = 0.037), pancreatic disease (5.8% vs 3.0%, *p* = 0.016), rectal cancer (3.9% vs. 0.8%) and esophageal cancer (8.5% vs 3.0%, *p* = 0.001) (Table 2).

### Trends in Postoperative Outcomes in Octogenarians

A decrease in LOS was observed in octogenarians who underwent surgery for colon cancer from median 9 days (IQR 6–14) in 2014 to 6 days in 2018 (IQR 4–11), ß − 0.54, 95% CI – 0.71 to − 0.36, *p* < 0.001) and rectal cancer (median 8 days (IQR 6–13.25) in 2014 to 6 days (IQR 5–10) in 2018, ß – 0.67, 95% CI – 1.09 to − 0.25, *p* < 0.001). In other quality registries, no difference in LOS was observed between 2014 and 2018.

A decrease in 30 day major morbidity between 2014 and 2018 was observed in octogenarians who underwent surgery for colon cancer (25.4% vs. 19.9%, OR 0.92, 95% CI 0.89–0.96, *p* < 0.001). No trends were observed in octogenarians regarding 30 day major morbidity in other quality registries.

No trends were observed in octogenarians regarding 30 day mortality between 2014 and 2018.

## Discussion

The current nationwide study on major surgical procedures in octogenarians did not show an increase in the proportion and absolute number of octogenarians that undergo surgery for esophageal cancer, stomach cancer, lung cancer, CRLM, pancreatic disease, colorectal cancer or AAA. For several major surgical procedures, postoperative outcomes such as LOS, 30 day major morbidity and 30 day mortality were worse in octogenarians. Improvement regarding LOS has been made after colorectal surgery in octogenarians. Postoperative morbidity and mortality did not improve during the study period in these patients.

Data from the Dutch Institute for Epidemiology show that aging of the population reached a plateau. An increase in octogenarians from 3.1% in 2000 to 4.3% in 2015 was observed while during the current study period an increase from 4.3% to 4.5% was observed [2]. In absolute numbers, this meant an increase from 717089 octogenarians in 2014 to 798820 octogenarians in 2018. The current study showed that no increase in the number of surgical procedures in octogenarians was observed in the past years. Several explanations are proposed such as earlier detection of malignancies due to early age surveillance and possibly stricter patient selection in multidisciplinary team meetings. Other reasons for no increase in octogenarians undergoing major surgical procedures might be new and less invasive multimodal alternatives for elderly patients such as definitive chemotherapy or (stereotactic) radiotherapy [21–24]. It was also described that a decrease in major surgical resection in elderly patients had taken place before the inclusion period of the current study [25]. Only for pancreatic cancer resection, a positive trend in the number of treated elderly patients was described [12]. It could be that we are now witnessing a plateau phase after a historical decrease in major surgical procedures in octogenarians. It is intriguing that this plateau phase has been witnessed in the current study as with current innovations such as less invasive surgery one would have expected to observe an increase in surgical procedures in octogenarians. However, with upcoming initiative such as prehabilitation, a future increase in the number of surgical procedures in octogenarians can possibly be expected.

Assessment of postoperative outcomes could aid clinicians in considering whether performing major surgical procedures is safe in octogenarians. Several postoperative outcomes were worse in octogenarians after esophageal cancer, stomach cancer, lung cancer, CRLM, pancreatic disease, colorectal cancer or AAA [12, 26–31]. This compares equally to the results in the current study. However, higher LOS might also reflect structural problems after surgery in octogenarians, such as problems regarding rehabilitation or transfer to a nursing home [32, 33]. The lower LOS of octogenarians who underwent AAA-repair can be attributed to the more frequent use of endovascular surgery in octogenarians [34]. As several factors influence LOS, other outcomes such as 30 day major morbidity and 30 day mortality better reflect outcomes in octogenarians. Unfortunately, only LOS improved after stomach and colorectal cancer surgery in the current study, while other postoperative outcomes did not improve for the various surgical fields. These observed decreases in LOS might be a result of the implementation of Enhanced Recovery After Surgery in these fields, but could also be attributed to the different patient selection per type of major surgical procedures. Measures to improve postoperative outcomes and therewith quality of care should be searched for by clinicians treating octogenarians. This study can be used as a benchmark to measure improvement of outcomes in elderly patients.

Results of the current study show that there is room for improvement regarding major surgical procedures in octogenarians. Preoperative selection should be (evidence) based on proper risk stratification and by making use of the optimalization of octogenarians by prehabilitation [35]. Using prehabilitation, modifiable risk factors can be optimized before surgery resulting in better postoperative outcomes [36]. As a result, it might be possible to perform major surgical procedures in more octogenarians due to more favorable outcomes resulting from implementation of multimodal prehabilitation [24]. Future studies should focus on this subject to improve quality of care for octogenarians undergoing major surgical procedures.

The current study has several limitations. First, the retrospective design of the study using registry data results in the lack of perioperative information and the differences between registries in the used outcomes can result in overestimation and underestimation of outcomes. Also, only surgically treated patients are registered in the several quality registries. No data are available on patients who were not surgically treated. Patient selection can therefore not be fully assessed in this study. Second, due to the inclusion of several large quality registries, no multivariable correction was performed when assessing postoperative outcomes. Assessment of the worse outcomes in octogenarians should therefore be assessed in depth by future studies on outcomes in separate quality registries. Third, a very recent inclusion period means that no information was available concerning the years before 2014. Before 2014, aging of the population probably increased significantly and it would have been interesting to have studied if a trend in surgical treatment of octogenarians and outcomes was observed. However, this study can be a benchmark for the coming years regarding trends and outcomes in octogenarians in various surgical fields.

In this study, it was shown that despite aging of the population, no increase in major surgical procedures in octogenarians was observed for esophageal cancer, stomach cancer, lung cancer, CRLM, pancreatic disease, colorectal cancer or AAA. Postoperative outcomes such as LOS and 30 day mortality, however, were worse in octogenarians for several major surgical procedures, and improvement is therefore warranted.

### Electronic supplementary material

Below is the link to the electronic supplementary material.

Supplementary file1 (JPEG 219 kb)

Supplementary file2 (DOCX 21 kb)

## Appendix 1

See Table 3

**Table 3:** STROBE statement—checklist of items that should be included in reports of *cohort studies*

	Item No	Recommendation
Title and abstract	1	(*a*) Indicate the study’s design with a commonly used term in the title or the abstract
		Done, page 1
		(*b*) Provide in the abstract an informative and balanced summary of what was done and what was found Done, page 1
*Introduction*		
Background/rationale	2	Explain the scientific background and rationale for the investigation being reported Done, page 2
Objectives	3	State specific objectives, including any prespecified hypotheses Done, page 2
*Methods*		
Study design	4	Present key elements of study design early in the paper Done, page ¾
Setting	5	Describe the setting, locations, and relevant dates, including periods of recruitment, exposure, follow-up, and data collection Done, page 3
Participants	6	(*a*) Give the eligibility criteria, and the sources and methods of selection of participants. Describe methods of follow-up Done, page 3
		(*b*) For matched studies, give matching criteria and number of exposed and unexposed Not a matched study
Variables	7	Clearly define all outcomes, exposures, predictors, potential confounders, and effect modifiers. Give diagnostic criteria, if applicable Done as far as outcomes, exposures is concerned, page 4 Rest not applicable for this study
Data sources/measurement	8*	For each variable of interest, give sources of data and details of methods of assessment (measurement). Describe comparability of assessment methods if there is more than one group Done, page 4
Bias	9	Describe any efforts to address potential sources of bias Done, in limitations (page 9)
Study size	10	Explain how the study size was arrived at Done, page 4
Quantitative variables	11	Explain how quantitative variables were handled in the analyses. If applicable, describe which groupings were chosen and why Done, page 5
Statistical methods	12	(*a*) Describe all statistical methods, including those used to control for confounding Done as far as applicable, page 5
		(*b*) Describe any methods used to examine subgroups and interactions Done, page 5
		(*c*) Explain how missing data were addressed Done, page 5
		(*d*) If applicable, explain how loss to follow-up was addressed Not applicable
		(*e*) Describe any sensitivity analyses Done, page 5
*Results*		
Participants	13*	(a) Report numbers of individuals at each stage of study—e.g., numbers potentially eligible, examined for eligibility, confirmed eligible, included in the study, completing follow-up, and analyzed Not applicable
		(b) Give reasons for non-participation at each stage Done (page 4 methods, and page 6 in total numbers)
		(c) Consider use of a flow diagram Was considered, but was not included as a result of conclusions by all authors
Descriptive data	14*	(a) Give characteristics of study participants (e.g., demographic, clinical, social) and information on exposures and potential confounders Done, Table 1 page 13/14
		(b) Indicate number of participants with missing data for each variable of interest Done, Table 1 page 13/14 (missing data patients were excluded)
		(c) Summarize follow-up time (e.g., average and total amount) Not applicable
Outcome data	15*	Report numbers of outcome events or summary measures over time
Main results	16	Give unadjusted estimates and, if applicable, confounder-adjusted estimates and their precision (e.g., 95% confidence interval). Make clear which confounders were adjusted for and why they were included Done, confounder adjusted estimates not applicable. Tables 1 and 2 page 13/14
		(*b*) Report category boundaries when continuous variables were categorized Not applicable
		(*c*) If relevant, consider translating estimates of relative risk into absolute risk for a meaningful time period Not applicable
Other analyses	17	Report other analyses done—e.g., analyses of subgroups and interactions, and sensitivity analyses Done (page 6/7)
*Discussion*		
Key results	18	Summarize key results with reference to study objectives Done page 8
Limitations	19	Discuss limitations of the study, taking into account sources of potential bias or imprecision. Discuss both direction and magnitude of any potential bias Done, page 9
Interpretation	20	Give a cautious overall interpretation of results considering objectives, limitations, multiplicity of analyses, results from similar studies, and other relevant evidence Done page 8/9
Generalizability	21	Discuss the generalizability (external validity) of the study results Done page 8/9
*Other information*		
Funding	22	Give the source of funding and the role of the funders for the present study and, if applicable, for the original study on which the present article is based Done, no funding (title page and last page)
