# Supplementary figures and images for: Outcomes After Major Surgical Procedures in Octogenarians: A Nationwide Cohort Study
Source: World J Surg. 2022 Aug 4;46(10):2399–408. doi: 10.1007/s00268-022-06642-6 (PMC9436861; doi:10.1007/s00268-022-06642-6)

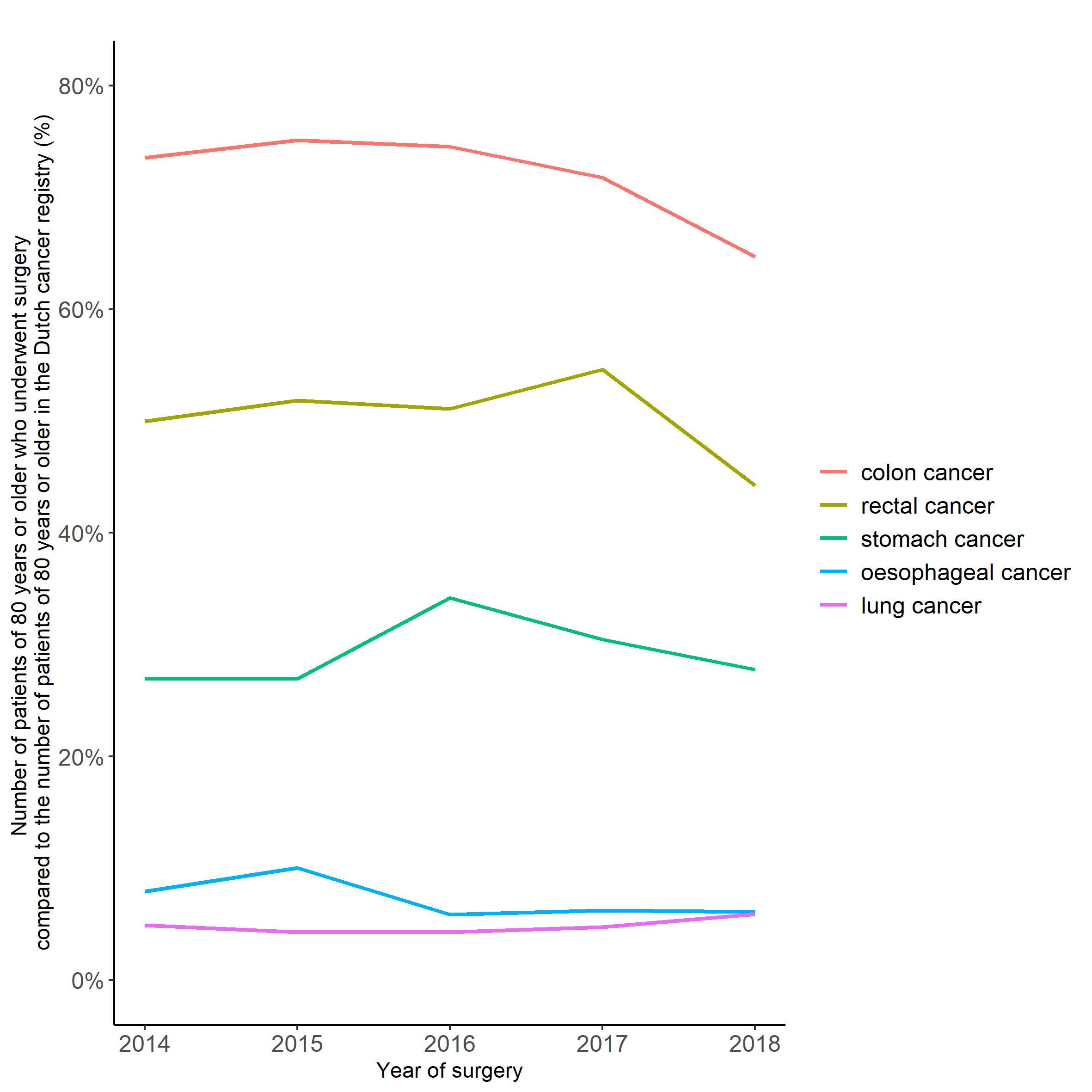

Supplement: Supplementary file 1 — Supplementary file1 (JPEG 219 kb) [file 268_2022_6642_MOESM1_ESM.jpg]
