# Supplementary material for: Outcomes After Major Surgical Procedures in Octogenarians: A Nationwide Cohort Study
Source: World J Surg. 2022 Aug 4;46(10):2399–408. doi: 10.1007/s00268-022-06642-6 (PMC9436861; doi:10.1007/s00268-022-06642-6)
Supplement: Supplementary file 2 — Supplementary file2 (DOCX 21 kb) [file 268_2022_6642_MOESM2_ESM.docx]

**Supplementary table 1.** Trend in proportion of surgically treated patients of 80 years or older per quality registry compared to the number of diagnosed patients of 80 years or older in the Dutch Cancer Registry (DCR).

| **Surgical treatment** | **2014** | **2015** | **2016** | **2017** | **2018** | **P-value trend** |
| --- | --- | --- | --- | --- | --- | --- |
| **Colon** |  |  |  |  |  |  |
| - **N – 80+ DCR: colon cancer** | 2124 | 1991 | 2033 | 1980 | 2047 |  |
| - **N - 80+ DCRA** | 1562 | 1496 | 1515 | 1421 | 1324 |  |
| - **% 80+ surgical treatment** | 73.5 | 75.1 | 74.5 | 71.8 | 64.7 | 0.116 |
| **Lung** |  |  |  |  |  |  |
| - **N – 80+ DCR: lung cancer** | 1867 | 2160 | 2119 | 2096 | 2134 |  |
| - **N – 80+ DCLA** | 92 | 93 | 91 | 100 | 126 |  |
| - **% 80+ surgical treatment** | 4.9 | 4.3 | 4.3 | 4.8 | 5.9 | 0.303 |
| **Stomach** |  |  |  |  |  |  |
| - **N – 80+ DCR: stomach cancer (excl. cardia tumors)** | 319 | 304 | 319 | 292 | 288 |  |
| - **N – 80+ DPCA** | 86 | 82 | 109 | 89 | 80 |  |
| - **% 80+ surgical treatment** | 27.0 | 27.0 | 34.2 | 30.5 | 27.8 | 0.669 |
| **Rectum** |  |  |  |  |  |  |
| - **N – 80+ DCR: rectal cancer** | 730 | 710 | 675 | 734 | 744 |  |
| - **N - 80+ DCRA** | 365 | 368 | 345 | 401 | 329 |  |
| - **% 80+ surgical treatment** | 50.0 | 51.8 | 51.1 | 54.6 | 44.2 | 0.553 |
| **Esophagus** |  |  |  |  |  |  |
| - **N – 80+ DCR: esophageal cancer** | 353 | 378 | 408 | 417 | 424 |  |
| - **N – 80+ DUCA** | 28 | 38 | 24 | 26 | 26 |  |
| - **% 80+ surgical treatment** | 7.9 | 10.1 | 5.9 | 6.2 | 6.1 | 0.221 |

N: number of patients

**Supplementary figure 1.** Trend in number of patients of 80 years or older who underwent surgery per quality registry compared to the total number of patients diagnosed per quality registry per year.*
